# Sensory functions in the foot soles in victims of generalized torture, in victims also beaten under the feet (falanga) and in healthy controls – A blinded study using quantitative sensory testing

**DOI:** 10.1186/1472-698X-12-39

**Published:** 2012-12-29

**Authors:** Karen Prip, Ann L Persson, Bengt H Sjölund

**Affiliations:** 1 Rehabilitation and Research Centre for Torture Victims, Copenhagen, Denmark; 2 Institute of Public Health, University of Southern Denmark, Odense, Denmark

**Keywords:** Chronic pain, Central inhibition, Falanga, Nerve injury, Sensitization, Torture

## Abstract

**Background:**

Falanga torture (beatings on the foot soles) produces local chronic pain and severe walking difficulties. We have previously reported signs of neuropathic pain in the feet of falanga victims. The objective here was to clarify underlying pain mechanisms by quantifying sensory impairments in the feet of torture victims who had experienced both generalized torture and those who had been exposed to falanga in addition. An ethnically matched control group was available.

**Methods:**

We employed quantitative sensory testing (QST) by investigators blinded to whether the patients, 32 male torture victims from the Middle East, had (n=15), or had not (n=17) been exposed to falanga. Pain intensity, area and stimulus dependence were used to characterize the pain as were interview data on sensory symptoms. QST included thresholds for touch, cold, warmth, cold-pain, heat-pain, deep pressure pain and wind-up to cutaneous noxious stimuli in the foot soles. Clinical data on anxiety and depression were retrieved.

**Results:**

Almost all falanga victims had moderate or strong pain in their feet and in twice as large an area of their foot soles as other torture victims. One-third of the latter had no pain in their feet and many reported slight pain; in spite of this, there were no differences in foot sole QST data between the tortured groups. A comparison with normal data indicated that both tortured groups had hypoesthesia for all cutaneous sensory fibre groups except those transmitting cold and heat pain, in addition to deep mechano-nociceptive hyperalgesia.

**Conclusion:**

A comparison of the QST data between victims having been exposed to generalized torture and victims who in addition had been exposed to falanga, showed no differences on the group level. The sensory disturbances in relation to our control group are compatible with central sensitization and de-sensitization, pointing to a core role of central mechanisms. A further analysis to create individual sensory profiles from our measurements is in progress.

## Background

The use of falanga (beatings on the soles of the feet) is a torture method which deliberately aims at inflicting intense pain in the feet and lower legs [1,2]. The method was originally used as legal punishment but is nowadays used more systematically as a torture method, especially in the Middle East and the Far East [3]. Years after torture the foot pain often persists [2,4,5] and contributes to severe disabilities [6], even when walking moderate distances. Also regarding generalized torture, the most common physical agent is unspecific beating [3,7]. In addition, it has been found that irrespective of torture method (physical or mental) the overall sequelae are similar [8].

The pathophysiological mechanisms of the chronic pain after falanga have puzzled clinicians and researchers. Some have hypothesized that a plantar closed compartment syndrome was the cause of pain and resulted in being unable to walk more than short distances [2,9,10]. Others have suggested a ruptured plantar aponeurosis [11], thickened fascia plantaris [12], or sustained bone trauma [13]. A reduction of the elasticity of the heel pads has also been proposed [11]. In single cases, fractures of metatarsal bones and aseptic bone necrosis have been reported [14,15].

Chronic pain is common among torture victims. Olsen at al. [7] found that more than 80% of patients referred for rehabilitation of torture sequaele reported chronic pain. Similar figures have been reported for war veterans with posttraumatic stress disorders (PTSD) [16]. In addition, Defrin et al. [17] recently found that persons with PTSD may have altered sensory processing with a combination of hypoesthesia and hyperpathia.

It is well known that nerve lesions may cause chronic neuropathic pain [18]. Thomsen et al. [19] examined 18 torture victims with severe pain to explore the origin of the pain generation using common bedside neurological assessment methods. They found a mixture of nociceptive and neuropathic pain conditions and a relation between specific neuropathic pain syndromes and exposure to four common types of torture (beatings all over the body, suspension, falanga and electric torture). Their most notable finding was the high prevalence of peripheral neuropathy in falanga victims.

In a group of persons exposed to falanga torture, we found [5] sensory dysfunction on clinical examination involving most sensory modalities in the feet compared to a non-tortured control group, confirming peripheral nerve lesions after falanga, including large as well as small sensory fibres. We concluded that signs of neuropathic pain were present in 10/11 victims and that the sensory findings indicated at least two neuropathic pain mechanisms: one dominated by a peripheral pain generator and the other by excitatory phenomena (dysaesthesia) indicating central sensitization [5].

Extending the observations that neuropathic conditions occur in the feet after falanga torture, the primary objective of this study was to help clarify the underlying pain mechanisms [5,20] by quantifying sensory impairments in the feet of two groups of torture victims, all referred for treatment at the Rehabilitation and Research Centre for Torture Victims in Copenhagen, Denmark. In addition, screening data on anxiety and depression were collected from the patient records. To fulfil the study objective, we employed quantitative sensory testing (QST) by investigators blinded to whether our torture victims had (F), or had not (NF) been exposed to falanga. We then compared the data between these two groups and to a separately recruited group of healthy men with no experience of torture from the Middle East.

## Methods

### Participants

The patients recruited were torture victims who had been granted asylum in Denmark. They were all referred to our centre from their general practitioner, because of their long-term sequelae from various types of torture that they had been subjected to several years earlier in their homeland. The patients were screened by an assessment team (physician, psychologist, physiotherapist and social worker, supported by an interpreter), with reference to the centre’s admission criteria: 1) torture victim with asylum in Denmark; 2) physical, psychological and social needs; 3) no overt psychosis; 4) no drug or alcohol abuse; and 5) available treatment capacity.

Following the main study, by snowball sampling, we managed to recruit 14 ethnically and age matched healthy men from the Middle East community in Copenhagen to form a control group, going through exactly the same QST methods as the patients. They had lived in Denmark for an average of 15.8 years (range 7–26), all spoke Danish and they were integrated and active in the Danish society. Their mean age was 37 years (range 21–55).

### Study selection criteria

**Figure 1:**
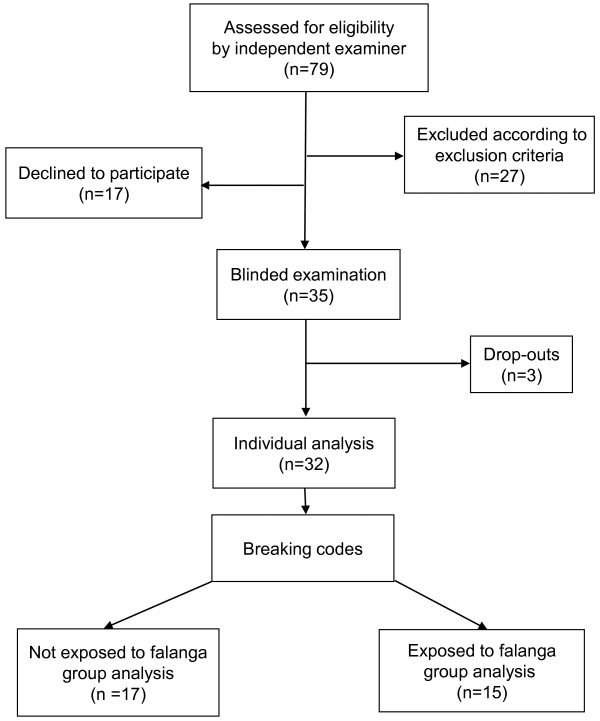
Flow chart showing the participants’ flow from eligibility to analysis.

The inclusion criteria for this study were tortured male patients originating from the Middle East speaking their native language, which was Arabic or Farsi (the majority of currently referred male patients during the period May 2009 – June 2010). Seventy-nine consecutively referred patients were identified via the electronic patient records (see flowchart in Figure 1). One senior physician (BHS) screened these medical records and excluded patients if: they had pathological structural changes in the feet and lower legs from reasons other than falanga, for example, fractures, amputations, extensive scar tissue after burning, cuttings or having foreign objects embedded in the feet/or having been wounded by foreign objects in the feet, such as shrapnel or bullets; nerve lesions in the lower legs from other reasons than falanga, for example, diabetic or alcoholic polyneuropathy and also injury to the central nervous system such as stroke or spinal fractures. Twenty-seven persons were excluded, usually due to injury to the nervous system other than from falanga. Patients were excluded because of: rhizopathy (n=7), diabetes (n=3), not being mentally fit (n=2), opioid medication (n=2), spinal fracture (n=1), arteriosclerosis in the legs (n=1), hydrocephalus (n=1), not been tortured but referred for having been secondarily traumatized (n=3), other problems and were referred for treatment elsewhere (n=7). Clinical symptoms and signs, including the typical temporal and anatomical progression of sensory symptoms of peripheral neuropathy were always sought for during the clinical history–taking and examination.

The remaining 52 patients were invited to participate in the study. At individual meetings the examiner (KP) informed them about the purpose and methods used in the study. If the patient agreed to participate an informed consent form was signed and dates for the assessments arranged. Seventeen turned down the offer, and three started but dropped out during the test sessions. Thus 32 patients participated in all three sessions. All participants were offered compensation for travel expenses. Arabic or Farsi interpreters assisted at all sessions. The project had been carefully introduced to the three interpreters involved, all of whom had long experience in interpreting for torture victims. Well informed about the procedures, they even volunteered for being tested with the techniques.

### Design

The examiner (KP) and a research assistant were blinded with regard to patient history, diagnosis and whether falanga torture had occurred or not. Breaking of the blinding took place only after the completion of all examinations and data analyses of the individual patients.

### Procedure

All examinations were scheduled to 3x2 hour sessions within a two week period and took place in a quiet room (stable temperature of 22–24°C) in the research department at our centre. For training the various QST methods were first practiced on healthy Danish volunteers.

We chose to perform the foot assessments on three different days to cause minimal discomfort for the patients. The 1^st^ session included an interview about pain and other sensory experiences, location and size of painful area [21] and foot pain intensity [22], testing of dynamic mechanical allodynia and dysesthesia in the feet [23]. At the 2^nd^ session tactile and thermal thresholds were examined [24-28], and at the 3^rd^ session pressure pain thresholds (PPTs; [28,29]) and wind-up pain upon repeated cutaneous mechano-nociceptive stimulation [17] were examined. The controls came only twice since the first session with the in-depth interview about pain was not necessary; furthermore, these persons were integrated in the Danish society and thus time consuming interpretation was not necessary.

In addition, psychological data were retrieved from the Hospital Anxiety and Depression Scale (HADS; [30]), in the patient records. The HADS was part of the psychological pre-assessment at our clinic. The patient was instructed to complete the questionnaire in order to record how he had felt during the past week. Each question was read by the psychologist in Danish, translated by the interpreter according to the Arabic or Farsi HADS version, respectively, and the patient marked his response on the relevant 0–3 Likert-scale. HADS consists of 14 statements that include two subscales. Seven questions are related to anxiety and seven to depression, each with a score range of 0–21. Scores 0–7 are regarded as normal and scores 8–10 are regarded as borderline cases (mild anxiety and depression) whereas scores above 11–14 (moderate) and 15–21 (severe) both indicate anxiety and/or depression.

At all foot examinations the participants lay on a couch with a special soft mattress. Individual needs were met to avoid painful body positions and make the patient as comfortable and relaxed as possible. Applications of the test stimuli were not visible to the patient at any time. All assessments and tests were performed by KP. A research assistant and an interpreter were also present.

### Test sites

To our knowledge only few studies have examined the arch of the foot sole with respect to normal QST values, and those existing are mostly recorded from the dorsum of the foot [26,31,32].

Five sites were identified by palpation for examining mechanical detection thresholds (MDT; [25]) and PPTs; [29]. They were encircled (10 mm in diameter) with a soft pen: one proximal to the first metatarso-phalangeal joint; one at the tuberosity of the calcaneum; one in the arch of the foot sole under the intermediate cuneiform bone, one at the lateral border of the sole (distal to the cuboid bone), and one proximal to the 5^th^ metarso-phalangeal joint. However, when the patients were asked which was the most painful area of their foot soles, they usually answered ‘under the arch’. Therefore, we chose the arch of the foot sole bilaterally for all remaining QST sensory tests. This site has been reported to be the most sensitive in the foot sole, both by our patients and in the literature [5,27].This choice also made comparisons between patients possible.

### Assessments

#### Interview

All participants were asked about pain in the foot soles at rest and when walking and the findings were registered on a 3-point Likert scale (0=no pain, 1=slight/moderate pain, and 2=severe pain; cf. [5]) to determine activity related changes in foot pain. From these data the victims’ feet could be divided into three groups: no foot pain; stimulus-independent foot pain (pain appearing spontaneously at rest); and stimulus-evoked foot pain (pain evoked by activity, such as when walking) [5]. Reported sensory disturbances such as numbness, cold/burning, pricking or buzzing sensations were also registered.

#### Pain drawings

Two pain drawings were used to assess pain locations [21]. The examiner (KP) asked the patient to shade in the locations of their pain on the surface of a body chart depicting the front and back of a human body and the painful areas of their feet on a special foot chart (views of right and left foot soles) [33]. In addition, the patients were asked to indicate the most painful region of their body and in both foot soles.The shaded-in areas on the pain drawings were measured in square millimetres and calculated in per cent of the total area using a commercial software program (Quantify One; K:L:O:N:K, Denmark), a method that has been shown to be reliable for quantifying pain drawings [34,35].

#### Pain intensity

Self-reported current pain intensity was assessed on a visual analogue scale (VAS; 0–100 mm; no pain=0; worst imaginable pain=100; [22]), with reference to the most painful body region and to each foot sole separately.

#### Quantitative sensory testing in the feet

We tested sensory modalities related to all major types of sensory nerve fibres using QST within the time constraints available for these vulnerable patients in both feet.

##### A. Mechanical detection thresholds

Tactile sensitivity under the foot soles was assessed bilaterally by measuring the mechanical detection threshold (MDT) to light touch using Semmes-Weinstein monofilaments (North Coast Medical, Inc.). We used 17 out of the 20 available monofilaments, from size 2.83 (target force 0.07 g) through to size 6.65 (target force 300 g). The detection threshold was defined as the least force that elicited a sensation of touch. The monofilament was applied at a 90° angle against the skin until it bowed during 1.5 s, held for 1.5 s and slowly released during 1.5 s [25]. The exact threshold was found by performing three repetitive tests with ascending fibre sizes, until one monofilament elicited at least one out of three responses (the participant saying ‘yes’). The next larger filament in size was applied to confirm the threshold. Regarding the patients, the detection thresholds were registered for all five sites, bilaterally. In the controls it was registered in the arch. The filament size registered was converted into target force in grams (g) according to a standardized conversion table.

##### B. Brush test

Dynamic mechanical allodynia is a painful or unpleasant sensation evoked by a cutaneous mechanical stimulus which does not normally evoke pain. To examine this phenomenon we used a qualitative test with light strokes with a soft brush (SENSELab^tm^– Brush-05; Somedic, Hörby, Sweden). Three consecutive strokes were applied with the brush to the skin in the arch of both foot soles over a 60 mm long distance [23] and the patient indicated if the stimulus was unpleasant or painful.

##### C. Thermal thresholds

The thermal tests [24,26,28,36] were performed to assess cold and warm detection thresholds (CDT and WDT) and cold and heat pain thresholds (CPT and HPT) using a TSA 2001 Peltier stimulator (MEDOC Inc., Israel). The Peltier thermode, size 3x3cm, was placed on the arch of the foot sole and attached with full contact to the skin using an elastic Velcro tape. We used the method of limits [24] and a baseline temperature of 32°C. The stimulator cut-off temperatures were set to 0°C for the cold and 50°C for the warmth assessments, respectively. If the participant did not respond to the stimulus before the cut-off limit was reached, this value was registered. The CDT and WDT were each measured by 4 ramped stimuli (1°C/s; return rate 1°C/s) and an inter-stimulus interval of 15 s. The CPT and HPT were also measured by 4 ramped stimuli (1.5°C/s; return rate 10°C/s) but with an inter-stimulus interval of 30 s. The participant was carefully informed about the procedure and instructed to press the push button the moment a cold/warm sensation was felt (sensory thresholds) and when the cold/warm stimuli became unpleasant or painful (pain thresholds). The detection thresholds used in the analyses were the mean values of the 4 stimuli for CDT, WDT, CPT and HPT, respectively. The skin temperature was measured bilaterally in the arch of the foot sole immediately prior to the thermal tests using a handheld laser FLUKE 62 mini IR thermometer with a distance of approximately 5 mm to the skin.

##### D. Pressure pain thresholds

Pressure pain thresholds (PPTs) were measured to assess deep mechanical nociception [37] by applying pressure to the five test sites bilaterally, for the controls the arch. We used an electronic pressure algometer (Somedic, Höör, Sweden; [29,38-40]). The algometer probe contact area was 10 mm and covered with 2 mm rubber. The instrument was calibrated to a zero level before each session. A pre-test was performed bilaterally on the radio-humeral extensor muscle group approximately 10 cm distal to the radio-humeral joint to familiarize the patient with the procedure. The most painful area reported in the foot soles was measured last in each series to avoid evoking discomfort. Always starting with the right foot sole, the examiner applied alternating series of three measurements on the five sites. A gradual pressure was applied vertical to the skin area and increased at a speed of 40 kPa/s controlled via monitoring on a display. The inter-stimulus interval was 30 s, and the inter-series interval 5 min. A cut-off was set at 900 kPa/s to avoid tissue damage [37]. The participant was instructed to press the push button when a sensation of pain or discomfort was perceived and the pressure ceased immediately. The PPT value, expressed in kPa/s, was the mean value of the last two sessions for each of the sites [39].

##### E. Temporal summation of mechano-nociceptive stimuli (wind-up pain)

Wind-up pain [41-44] refers to central pain sensitization caused by repeated painful stimulation of peripheral nerves at sufficient intensity to stimulate C-fibres, leading to progressively increasing response in the corresponding spinal posterior horn neurons. For this measure we used the thickest available Semmes-Weinstein monofilament (size 6.65; [45]). At 0.3 Hz the examiner applied the filament four consecutive times to the surface of the skin in the arch of each foot sole. The patients were asked to rate the pain intensity on a VAS after the 1^st^ and 4^th^ stimulus. A 5-minute pause followed. Thereafter, to produce a more intense stimulation, 10 consecutive stimuli were applied at the rate of 1.0 Hz. The patients rated their pain after the 1^st^ and 10^th^ stimulus. If the VAS difference between the 1^st^ and last stimulus was positive, a temporal summation (wind-up) had occurred.

### Statistics

We chose to analyse the data from the right and left feet separately, since they may share common analysis mechanisms in the central nervous system, even if separate. The mean, SD, 95% CI, median, and range were calculated for all ten variables, as were the correlations between both feet. Students t-test or Fisher’s exact test was used to test for differences between sides.

To detect differences between the torture groups and the controls, identified after breaking the blinding as victims not exposed to falanga (NF) and victims exposed to falanga (F), we used univariate ANOVA’s test. A *post hoc* Tukey analysis was performed to indicate the differences between data in the respective groups. Acceptable p-values were set to ≤0.05.

The data were analysed using the Statistical Package for Social Sciences (SPSS) version 18, Software for Windows (SPSS, Chicago; IL; USA).

### Ethics

Each participant was informed verbally about the study and was also given an information kit containing a comprehensive written description translated into their respective languages. They also received translated guidelines concerning participation in medical research issued by the Danish Ethical Committee. The assessments comply with the Helsinki II Declaration [46] and the patients could withdraw from the study at any time, without any impact on their planned rehabilitation at our clinic. The study was approved by the Research Ethics Committee in Region Copenhagen, Denmark (H-D-2009-068) and registered at the Danish Data Protection Agency.

## Results

### Patient characteristics

Figure 1 illustrates that 79 consecutive patients were identified as eligible to participate in the study. Twenty-seven patients were excluded due to: rhizopathy (n=7), diabetes (n=3), not being mentally fit (n=2), opioid medication (n=2), spinal fracture (n=1), not tortured but referred for having been secondarily traumatized (n=3), arteriosclerosis in the legs (n=1), hydrocephalus (n=1), or referred for treatment elsewhere (n=7). Seventeen declined to participate and three dropped out; thus 32 patients participated in all three test sessions. When breaking the blinding, it turned out that 17 patients had not been exposed to falanga (NF) and 15 had been exposed to falanga (F). Of the 20 patients that declined to participate or dropped out, 9 had been exposed to falanga.

The mean age did not differ between the two groups: NF = 44.5 years (range 34–63) and F = 46.3 years (range 38–55). The NF victims had been tortured for the first time during the years 1979–2000 and had spent a median of 180 days (range 1–2372) in prison, whereas the F victims had been tortured for the first time during the years 1989–1996 and had spent a median of 365 days (range 15–1700) in prison.

All 32 patients reported pain in many parts of the body, located in at least 3 out of 4 quadrants. When asked about their one most painful region, neck, shoulder and low-back pain were the most common complaints, and only two patients in the F group reported their foot pain as being the most severe. The mean body pain area size in the NF patients was 13.8% of the total body area (median 11%; range 2-39%) whereas for the F patients it was 21.0% (median 14.0%; range 4-57%). The mean current body pain intensity (VAS) in the NF patients was 48 mm (median 44; range 15–100 mm) whereas for the F patients it was 60 mm (median 55; range 23–100 mm). Neither of these differences was significant (Student’s t-test).

Since we could collect data from 64 feet but the central pain processing occurred in 32 persons, we examined whether QST data from the two sides of a single individual corresponded. It turned out that the Pearson correlation coefficient from the right versus left data of the torture victims was generally high (Table 1) and the t-test did not show any significant difference between the sides. The same held for the control data (Table 1).

**Table 1:** **Right vs**. **left feet similarities regarding pain characteristics and sensory functions for the 32 torture victims and the 14 controls**

	**Pain intensity VAS**	**Pain area %of foot sole**	**MDT target force**	**CDT**	**WDT**	**CPT**	**HPT**	**PPT**	**Wind**-**up at 0.3 Hz**	**Wind**-**up at 1.0 Hz**
**Torture victims** right/left feet										
Pearson correlation (p-value)	0.798 (<0.001)	0.730 (<0.001)	0.525 (0.002)	0.847 (<0.001)	0.783 (<0.001)	0.809 (<0.001)	0.738 (<0.001)	0.818 (<0.001)	0.036 (0.053)	0.724 (<0.001)
Student’s t-test *p*-value	0.104	0.229	0.108	0.386	0.281	0.413	0.315	0.529	0.215	0.088
**Controls** right/left feet										
Pearson correlation (p-value)			0.841 (<0.001)	0.824 (<0.001)	0.550 (0.041)	0.817 (<0.001)	0.726 (0.003)	0.920 (<0.001)	0.643 (0.013)	0.944 (<0.001)
Student’s t-test p-value			0.170	0.897	0.312	0.065	0.646	0.147	0.981	0.158

VAS, visual analogue scale; MDT, mechanical detection threshold; CDT, cold detection threshold; WDT, warm detection threshold; CPT, cold pain threshold; HPT, heat pain threshold; PPT, pressure pain threshold; Wind-up mm at 0.3 Hz (mean pre/post difference in pain intensity VAS mm after wind-up stimulation at 0.3 Hz); Wind-up mm at 1.0 Hz (mean pre/post difference in pain intensity VAS mm after wind-up stimulation at 1.0 Hz).

It was possible to retrieve data on anxiety and depression in 26 out of our 32 patients (NF=12; F=14) from HADS forms. The median anxiety score in NF victims was 19.5 points (11 out of 12 patients had ≥ 15 points) and in F victims was 19.0 points (13 out of 14 patients had ≥ 15 points) demonstrating severe anxiety in both groups. The median depression score in NF victims was 16.5 points (8 out of 12 patients had ≥ 15 points) ) and in F victims was 17 points (10 out of 14 patients had ≥ 15 points), likewise pointing to severe depression.

### Pain conditions in the feet

#### Pain characteristics

The 32 patients were categorized according to their reported activity related foot pain when walking. Seven feet (NF/F: 4/3) exhibited stimulus-independent foot pain, whereas stimulus-evoked pain was perceived in 45 feet (NF/F: 20/25). No pain was reported in 12 feet (NF/F: 10/2). Thus only 2/30 F feet were pain free whereas 10/34 NF feet were so (p=0.062; Fisher’s exact test). A corresponding comparison between pain free feet and those with stimulus-evoked pain (NF/F: 10/2 vs. 20/25) was barely significant (p=0.023; Fisher’s exact test).

Current foot pain intensity at rest was ≤ 30 mm (VAS ‘slight pain’; [47]) in 20/34 feet of the NF patients whereas 17/28 patients in the F group had moderate or severe pain (VAS > 30 mm). The mean pain area covered 21% of the foot sole in the NF group; however, in the F group it was 44%, a highly significant difference (p<0.001; Student’s t-test).

#### Reported sensory disturbances

From the interviews regarding the 64 feet (relation NF/F 34/30 feet), numbness was experienced in 21/14 feet; cold sensation in17/12 feet; burning sensation in 20/22 feet and a pricking sensation in 17/20 feet, respectively.

The brush test (NF/F) demonstrated dysesthesia in 15/14 feet, and dynamic mechanical allodynia in 4/2 feet. Thus, there were no obvious differences between those exposed to falanga or not regarding irritative phenomena.

#### QST in the feet

As can be seen in Tables 2 and 3 an ANOVA analysis with Tukey’s post hoc test of all data showed no significant QST differences between the two torture groups. However, in relation to the control values there was significant hypoesthesia for mechanical thresholds (MDT) and hyperalgesia for deep mechanical nociception (PPT) in the two groups, whether exposed to falanga or not. Cold (CDT) and warm detection (WDT) were significantly impaired compared to controls but only in the no falanga group (Tables 2 and 3).

**Table 2:** **Sensory function in the right foot of 15 falanga** (**F**) **and 17 no falanga** (**NF**) **torture victims and 14 healthy controls** (**C**) (**mean**, **SD**, **95**% **CI**, **median and range for eight QST variables**)

**Right foot**	**MDT**	**CDT**	**WDT**	**CPT**	**HPT**	**PPT**	**Wind**-**up mm**	**Wind**-**up mm**
	**target force g**	**°C**	**°C**	**°C**	**°C**	**kPa**	**at 0.3 Hz**	**at 1.0 Hz**
	**Mean**	**Mean**	**Mean**	**Mean**	**Mean**	**Mean**	**Mean**	**Mean**
	**SD**	**SD**	**SD**	**SD**	**SD**	**SD**	**SD**	**SD**
	**CI**	**CI**	**CI**	**CI**	**CI**	**CI**	**CI**	**CI**
	**Median**	**Median**	**Median**	**Median**	**Median**	**Median**	**Median**	**Median**
	**(Range)**	**(Range)**	**(Range)**	**(Range)**	**(Range)**	**(Range)**	**(Range)**	**(Range)**
Falanga n=15	5.66	25.2	43.2	19.1	47.0	252	16	29
	15.22	4.8	4.9	9.2	4.6	206	20	23
	−2.77–14.09	22.5–27.9	40.5–45.9	14.0–24.2	44.5–49.6	138–365	4–28	16–43
	1.00	27.0	44.5	21.7	49.9	181	10	27
	(0.16–60.00)	(14.4–30.7)	(35.5–50.0)	(0–29.2)	(37.9-50.0)	(19–833)	(−3–72)	(0–82)
No Falanga n=17	3.71	22.5	44.2	15.3	48.9	275	5	27
	4.62	7.7	4.7	9.7	1.8	205	8	18
	1.33–6.09	18.4–26.5	41.7–46.7	9.9–20.6	47.9–49.9	169–380	1–9	17–36
	1.00	24.8	44.6	18.5	50.0	214	2	35
	(0.07–15.00)	(0–29.4)	(34.0–50.0)	(0–26.3)	(44.3–50.0)	(98–818)	(−4–23)	(0–51)
Controls n=14	0.41	28.0	40.1	18.2	46.7	485	17	30
	0.35	2.4	2.8	8.3	2.5	179	14	19
	0.21–0.61	26.6–29.4	38.5–41.7	13.5–23.0	45.3–48.2	381–588	9–25	18–41
	0.28	28.8	40.3	21.2	46.8	460	17	28
	(0.07–1.00)	(20.9–30.1)	(35.3–43.3)	(2.6–27.7)	(42.4–50.0)	(240–912)	(−3–45)	(0–62)
ANOVA *p*-*value*	**0**.**010** *	**0**.**019**	**0**.**030**	0.505	0.150	**0**.**005**	**0**.**050**	0.924
*Tukey*’*s post hoc test* F/NF	0.880 *	0.278	0.791	0.507	0.264	0.941	0.122	0.949
F/C	**0**.**043** *	0.361	0.128	0.967	0.964	**0**.**008**	0.970	0.999
NF/C	**0**.**011** *	**0**.**014**	**0**.**027**	0.673	0.176	**0**.**014**	0.067	0.930

MDT, mechanical detection threshold; g, gram; CDT, cold detection threshold; WDT, warm detection threshold; CPT, cold pain threshold; HPT, heat pain threshold; PPT, pressure pain threshold; kPa, kiloPascal; Wind-up mm at 0.3 Hz (mean pre/post difference in pain intensity VAS mm after wind-up stimulation at 0.3 Hz); Wind-up mm at 1.0 Hz (mean pre/post difference in pain intensity VAS mm after wind-up stimulation at 1.0 Hz); * calculated on log MDT.

**Table 3:** **Sensory function in the left foot of 15 falanga** (**F**) **and 17 no falanga** (**NF**) **torture victims and 14 healthy controls** (**C**) (**mean**, **SD**, **95**% **CI**, **median and range for eight QST variables**)

**Left foot**	**MDT**	**CDT**	**WDT**	**CPT**	**HPT**	**PPT**	**Wind**-**up mm**	**Wind**-**up mm**
	**target force g**	**°C**	**°C**	**°C**	**°C**	**kPa**	**at 0.3 Hz**	**at 1.0 Hz**
	**Mean**	**Mean**	**Mean**	**Mean**	**Mean**	**Mean**	**Mean**	**Mean**
	**SD**	**SD**	**SD**	**SD**	**SD**	**SD**	**SD**	**SD**
	**CI**	**CI**	**CI**	**CI**	**CI**	**CI**	**CI**	**CI**
	**Median**	**Median**	**Median**	**Median**	**Median**	**Median**	**Median**	**Median**
	**(Range)**	**(Range)**	**(Range)**	**(Range)**	**(Range)**	**(Range)**	**(Range)**	**(Range)**
Falanga n=15	33.98	24.4	43.5	20.6	47.6	239	6	26
	86.70	7.6	4.1	7.3	3.9	142	21	25
	−14.03–82.00	20.2–28.7	41.2–45.8	16.5–24.6	45.5–49.8	160–317	−7–19	11–42
	1.40	27.9	43.8	20.2	49.5	209	10	20
	(0.16–300.00)	(0–30.1)	(36.0–50.0)	(0–29.1)	(36.5–50.0)	(78–488)	(−50–35)	(0–78)
No Falanga n=17	9.47	21.9	45.1	15.5	49.2	262	6	20
	16.17	7.5	3.5	9.6	1.4	179	12	16
	1.16–17.79	17.9–25.9	43.2–46.9	10.2–20.8	48.4–50.0	170–353	−1–12	12–29
	2.00	24.3	45.5	19.7	50.0	210	3	21
	(0.07–60.00)	(0–29.4)	(39.1–50.0)	(0–27.8)	(45.0–50.0)	(94–745)	(−14–29)	(−1–50)
Controls n=14	0.33	28.0	40.9	21.1	47.0	456	17	27
	0.34	2.4	3.1	4.7	2.5	167	13	22
	0.14-0.53	26.6–29.3	39.1–42.7	18.3–23.8	45.5–48.4	359–552	10–24	14–39
	0.16	28.9	41.3	20.6	47.6	461	18	26
	(0.07–1.00)	(21.6–30.2)	(36.1–45.1)	(9.2–27.9)	(42.0–49.8)	(208–783)	(0–39)	(−2–63)
ANOVA *p*-*value*	**0**.**002** *	**0**.**045**	**0**.**011**	0.099	0.093	**0**.**001**	0.096	6.656
*Tukey*’*s post hoc test* F/NF	0.919 *	0.518	0.444	0.172	0.278	0.918	0.996	0.725
F/C	**0**.**003** *	0.314	0.143	0.983	0.794	**0**.**003**	0.163	1.000
NF/C	**0**.**007** *	**0**.**035**	**0**.**008**	0.130	0.088	**0**.**006**	0.123	0.701

MDT, mechanical detection threshold; g, gram; CDT, cold detection threshold; WDT, warm detection threshold; CPT, cold pain threshold; HPT, heat pain threshold; PPT, pressure pain threshold; kPa, kiloPascal; Wind-up mm at 0.3 Hz (mean pre/post difference in pain intensity VAS mm after wind-up stimulation at 0.3 Hz); Wind-up mm at 1.0 Hz (mean pre/post difference in pain intensity VAS mm after wind-up stimulation at 1.0 Hz); * calculated on log MDT.

Immediately before thermal testing the plantar skin temperature was measured and the mean was found to be 30.8°C (NF) and 30.4°C (F), similar to values found in the literature [48]. During the measurements we had to stop the thermal test for two NF victims, once for psychological reasons and once because the pain became unbearable, spreading upwards in both lower legs. This pain condition lasted into the following day, probably due to temporal summation.

The mean pre/post difference in pain intensity for the F group after repeated cutaneous mechanical stimulation at 0.3 Hz (cf. [17]) was 16 mm for the right foot and 6 mm for the left foot, twice that of the NF group (5–6 mm) but not, however, statistically different. A more intense “wind-up” stimulation with 10 stimuli at 1.0 Hz produced about equal temporal summation in both groups (VAS mean pre/post difference for F=29 mm and for NF=27 mm for the right foot and for the left foot 26–20 mm). However, the wind-up effect was about the same in both feet of the controls as in the falanga group (Tables 2 and 3).

#### Possible influence of the mental state

Using correlation analysis we also explored other variables to explain our findings and found a clear correlation between the severity of HADS anxiety score and foot pain intensity (all feet; r=0.44; p=0.001; Spearman’s test). Regarding body pain intensity, only a tendency to correlation was seen (r=0.38; p=0.059). The same was found for body pain area size (r=0.39; p=0.047). There were no correlations between body pain intensity/body pain area size and the HADS depression scores. Regarding the QST variables, there were no significant correlations to HADS scores at all.

## Discussion

### Pain and sensory function

We compared the sensory findings from victims exposed to generalized torture to victims who had additionally been exposed to falanga, as an extension to earlier studies from our centre [5,6,19]. In addition, we examined an ethnically matched healthy control group with the same QST tests for comparison. After breaking the blinding, the share of victims not exposed to falanga (NF) and victims exposed to falanga (F) turned out to be about the same (Figure 1). This finding was unexpected, since from earlier studies [6], most torture victims from the Middle East have been beaten under the feet [3].

In spite of the expected clinical differences in foot pain symptoms between the two tortured groups, our main observation was that there were no significant differences between them regarding the QST findings. However, in comparison to our control data, the picture is instead dominated by a generalized hyposensitivity to non-noxious cutaneous stimuli in the foot soles in combination with deep mechanical hyperalgesia. A possible interpretation of these unexpected findings could be that the impact of generalized torture is so heavy on the central nervous system that the situation of the individual is dominated by a global change in central function. This would consist of a blend of sensory sensitization (deep mechanical hyperalgesia; [49]), de-sensitization (cutaneous hypoesthesia; [17]) and mental changes like strong anxiety and depression. It should be remembered that patients are referred to our centre because they experience physical, psychological and social sequelae from torture. Hence, many of them (56%; [50]) suffer from complex PTSD or ‘disorders of extreme stress not otherwise specified’ (DESNOS; [51]) which may indeed influence central nervous system function [17]. Thus, the influence of the local trauma against the foot soles (falanga) seems to be too small to be detected by the QST techniques under these circumstances.

The elevated MDTs indicate that both groups had reduced protective sensation. According to Bell-Krotoski [25] the 2.83 filament (target force 0.07 g) is optimal for detection of mechanical stimuli in most body areas with the exception of the foot sole, where a slightly stronger filament 3.61 (0.4 g) is recommended for normal subjects. We had to use a much stronger filament to obtain a response indicating hypoesthesia due to a partial loss of function in the Aβ-fibres [28,52].

HPTs and CPTs were within normal range in both groups of torture victims. Regarding PPTs, we found hyperalgesia to deep mechanical stimuli in both groups, probably due to a central sensitization, or possibly due to peripheral sensitization of C-fibre nociceptors [37].

In a sense, our QST findings comply well with the results of researchers studying other neuropathic pain conditions. Their study results have been found to comprise a complex web of different pain and sensory characteristics [49,53-57] rather than consistent findings related to a particular causative factor, in our case a repetitive mechanical trauma. In the clinical situation it may therefore be important, as recommended by several authors [52,57-59] to examine each person’s sensory profile as various sensory symptoms differ between individuals.

The only previous study of chronic pain in patients suffering from PTSD was conducted by Defrin et al. [17]. They found a higher prevalence of chronic pain compared to a group with common anxiety disorder and a significant correlation between chronic pain intensity and PTSD severity. This observation fits with our finding of a significant correlation between the HADS anxiety scores and foot pain intensity (VAS). Furthermore, they found that QST revealed higher MDT, WDT and HPT and lower CDT in the PTSD cases compared to those with anxiety disorder and normal subjects. Their three groups (PTSD, anxiety disorders and healthy controls) exhibited a temporal summation following mechanical stimulation at 0.3 Hz, but the PTSD subjects scored slightly higher. Conversely, our patients showed little or control-size wind-up when using the parameters of Defrin et al., which could be due to central changes. Defrin et al. [17] attributed their findings to the manner in which PTSD subjects emotionally interpret and respond to pain stimuli. At variance with this interpretation, we found little or no correlation between pain characteristics and QST data and the degree of depression or anxiety in our patients. It is reasonable to assume that all our patients had sustained a combination of physical and psychological trauma producing a less clear picture.

### Study limitations

#### Regarding the study subjects

The patients were refugees with residence permit in Denmark and referred by their general practitioner to our specialized clinic, making our sample highly selected and therefore probably not representative of all torture victims.

With our traumatized patients, it was not possible to collect exact information on the extent of falanga torture, that is, how often and how severely the victims had been beaten. Attempts to retrieve such information may produce intense anxiety and flash backs. Moreover, all patients had been subjected to various forms of torture, usually during extended periods of detention, increasing the risk of brain injuries [60], which may contribute to the pain reported. Torture victims are vulnerable, often forget and have difficulties to focus attention [51]. Using QST requires cooperation from the patient and the dimensions of cognitive effects on QST findings are not resolved [61,62]. Indeed, there are recent indications that chronic widespread pain is associated with lower cognitive processing speed [63]. The psychophysical nature of the QST data may have been influenced by the high levels of anxiety and depression present in our patients. However, we found no correlations to single QST variables and the consistent mixture of hypoesthesia and hyperalgesia speaks against such a general effect.

Since many of these patients have been imprisoned for long time periods, it is important to distinguish the present findings from those elicited by peripheral neuropathy, whether from toxic, nutritional or infectious causes. However, the victims were carefully examined for such comorbidities during the medical assessment (see Methods) and secondly, motor deficits, typical for severe polyneuropathies, were never found among the included patients. Furthermore, the combination seen in our patients, cutaneous hypoesthesia, normal nociceptive transmission and deep mechanical hyperalgesia, is not typical for peripheral neuropathies but has been reported in, for example, chronic regional pain syndrome (Appendix A in [49]). Nevertheless, a component of sensory neuropathy in the torture victims cannot be completely excluded.

#### Regarding the QST techniques

Recently there has been a debate on whether QST is of value in assessing sensory disturbances in single patients [58,64]. The consensus seems to be that QST, although demonstrating a high specificity, has a low sensitivity that does not always pick up discrete sensory abnormalities as well as the clinical examination does. In the present study it cannot be ruled out that minor signs of nerve injury in the foot soles were not detected by our QST measurements, which may explain the discrepancy with our previous clinical study [5]. It may also be that individual variations make the heterogeneity within groups too big to demonstrate group differences. We will therefore continue the QST analysis by producing individual sensory profiles for all participants, containing both sensory symptoms and QST data [49,53,57,58,65,66].

## Conclusion

In conclusion, a comparison of the QST data between victims having been exposed to generalized torture and victims who in addition had been exposed to falanga, unexpectedly showed no differences at the group level. This was so even though almost all the falanga victims had moderate to strong pain in their feet and in twice as large an area of their foot soles as the torture victims not exposed to falanga. One-third of the latter did not report pain in their feet at all and many reported only slight pain. On the other hand, the comparison to our normal data indicated that there was hyperalgesia to deep mechano-nociceptive stimuli, irrespective of exposure to falanga or to other forms of torture. In addition, the cutaneous sensory fibre groups, except those transmitting cold and heat pain, were less sensitive to external stimuli. The findings are compatible with central sensitization and de-sensitization, pointing to a core role of central mechanisms. One way to strengthen the analysis is to create sensory profiles from our data on the individual level, which has been done in the subsequent paper [66].

## Competing interests

The authors declare that they have no competing interests.

## Authors’ contributions

KP has been active in all aspects of the study: conception, design, acquisition of data (main responsibility), analysis and interpretation of data, writing of the manuscript and final approval. ALP contributed to the conception, design, analysis, and interpretation of data (main responsibility), writing of manuscript and final approval. BHS had main responsibility for the conception and design of the study, contributed to the interpretation of data and participated in writing of manuscript and final approval. All authors read and approved the final manuscript.

## Authors’ information

KP, PT, MSc, has long experience of treating victims of torture since more than twenty years at our centre. This research is intended to become part of her PhD - thesis. ALP, PT, PhD, is a Senior Researcher in pain and rehabilitation research at our centre and has extensive experience of multidisciplinary pain rehabilitation. BHS, MD, DMSc, Professor of Rehabilitation at the University of Southern Denmark, is a pain management physician and was till recently Director General of our centre.

## Pre-publication history

The pre-publication history for this paper can be accessed here:

http://www.biomedcentral.com/1472-698X/12/39/prepub
